# Gut microbiota profiles of young South Indian children: Child sex-specific relations with growth

**DOI:** 10.1371/journal.pone.0251803

**Published:** 2021-05-14

**Authors:** Nirupama Shivakumar, Ambily Sivadas, Sarita Devi, Farook Jahoor, John McLaughlin, Craig P. Smith, Anura V. Kurpad, Arpita Mukhopadhyay

**Affiliations:** 1 Division of Nutrition, St. John’s Research Institute, St. John’s National Academy of Health Sciences, Bangalore, India; 2 Department of Pediatrics, USDA/Agricultural Research Service Children’s Nutrition Research Center, Baylor College of Medicine, Houston, Texas, United States of America; 3 Faculty of Biology, Division of Diabetes, Endocrinology and Gastroenterology, School of Medical Sciences, Medicine & Health, Manchester Academic Health Sciences Centre, University of Manchester, Clinical Sciences Building, Salford Royal Hospital, Salford, United Kingdom; 4 Department of Physiology, St. John’s Medical College, St. John’s National Academy of Health Sciences, Bangalore, India; Wageningen Universiteit, NETHERLANDS

## Abstract

Gut microbiota has been implicated as a modifier of childhood growth. Here, 16S rRNA sequencing-based fecal microbiota profiles of 18–24 month old Indian children were evaluated (n = 41), in relation to their anthropometric parameters, intestinal permeability, body composition and total energy expenditure. Pathway analyses were conducted to assess microbial functions related to stunting, underweight and wasting. The fecal microbiota was enriched in *Prevotella 9*, *Bifidobacterium* and *Escherichia-Shigella*. Weight, weight-for-age Z-scores (WAZ) and weight-for-length Z-scores (WLZ), along with age, acted as covariates of microbiota variation specifically in boys (n = 23). *Bifidobacterium longum subsp longum* abundance was positively associated with WAZ while *Bifidobacterium bifidum* and *Bifidobacterium breve* abundances were negatively associated with age. The lipopolysaccharide biosynthesis pathway was upregulated in stunted (n = 16) and wasted (n = 8) children. Findings from this study indicate that child sex may be a critical modifier of the role of gut microbiota on childhood growth.

## Introduction

Suboptimal nutritional status is prevalent in children under 5 years of age in low- and middle-income countries despite noticeable progress in regions such as Central and tropical America [1]. Due to considerable disparities in socioeconomic and demographic factors amongst the low and middle income countries, the WHO Global Nutrition Targets set in 2010 to reduce stunting by 40% and wasting to <5% by 2025 are unlikely to be achieved [1]. Achieving these targets requires efforts to first identify underpinning mechanistic pathways followed by relevant interventions to promote child growth.

Prevailing nutritional interventions have barely been able to improve child growth faltering. A contributing factor toward this conundrum could be the failure of the intervention regimes to address the role of the gut in child growth faltering. Specifically, the role of environmental enteropathy (EE) or Environmental Enteric Dysfunction (EED) in growth faltering per se and also in recovering from faltered growth due to lack of appropriate response to nutritional therapies, has gained a lot of attention [2,3]. Accumulating evidence suggests that child growth may be compromised by a complex interplay (host-microbiome interactions) between the resident microbiota, intestinal integrity, immune response and food intake and metabolism on child growth [4].

A recent study has established a causal pathway relating duodenal microbiota, enteropathy and stunted growth in children from urban slums of Bangladesh [5]. Pre-clinical models that have attempted to provide links between undernourishment, intestinal pathogenic insult and EE, indicate the possibility of varying EE phenotypes that could depend on a combination of diet deprivation and pathogens [6]. This has been confirmed by associations between poor nutritional status and altered fecal microbiome in children from India and elsewhere [7–10]. However, intervention with specially designed microbiome directed complementary foods do not have a faster rate of recovery from moderate acute malnutrition compared to routinely provided ready-to-use therapeutic foods [11]. Considering the known links between geographical location, dietary habits and gut microbiome profiles [12,13], it is also necessary to advance existing knowledge by conducting in-depth characterization of gut microbiome profiles of undernourished children from diverse geographical areas. The current study evaluated the hypothesis that the fecal microbiome profiles of 41 young Indian children aged 18–24 months is associated with their anthropometric, demographic, body composition and intestinal permeability variables aligned to their nutritional and EE status.

## Materials and methods

### Participant details

The selection of participants for this study (n = 41, 18–24 months age) was based on the availability of fecal samples for gut microbiota analysis from a larger study for which apparently healthy children had been recruited from an urban slum in close proximity to St. John’s National Academy of Health Sciences to determine tryptophan metabolism and protein digestibility in children at risk of EE. The study had been approved by the Institutional Ethics Committee, St. John’s Medical College and Hospital, Bangalore, India and written informed consent had been obtained from caregivers of the participants. The study was registered at the Clinical Trials Registry of India (http://ctri.nic.in/Clinicaltrials/login.php); registration number: CTRI/2017/02/007921.

Non-breastfed children, aged between 18–24 months, underwent a pediatric clinical examination and history taking to exclude chronic or congenital systemic disorders, history of food allergies or serious illness in the past three months. Children with a history of gastrointestinal symptoms in the previous two weeks, antibiotic therapy in the last four weeks, and regular iron supplementation were excluded. A screening blood sample was collected to test for C-reactive protein (CRP), HIV, Hepatitis B and hemoglobin (Hb) (ABX-Pentra 60C+, France). Information on general demographic characteristics were collected through caregiver interviews. Anthropometric measurements were performed employing standard procedures and equipment, weight was measured to the nearest 10 g (Seca 354, Hamburg, Germany) and length to the nearest millimeter (Seca 417, Hamburg, Germany). Nutritional status indicators, length-for-age Z-scores (LAZ), weight-for-age Z-scores (WAZ) and weight-for-length Z-scores (WLZ) had been extracted using the WHO anthroplus software (version 3.2.2, January 2011). Based on established cut-offs for nutritional indicators [14], the participants were categorized as stunted (LAZ < -2), underweight (WAZ < -2) or wasted (WLZ < -2), respectively.

### Body composition, total energy expenditure, dual sugar assay measurements and calculations

For body composition and total energy expenditure (TEE) measurements, the participants drank doubly labelled water (DLW), after ensuring 2 hour post-prandial period, at a dose of 0.2 g/kg body weight (BW) of 99.9% ^2^H_2_O and 3 g/kg BW of 10% H_2_^18^O (supplied by Sercon Ltd, Cheshire, UK). A basal urine sample was collected before dosing, at home by the mothers into a sterile container, and one sample between 3 to 4 h post DLW dose for body composition, and another sample after 1 week (at home) for TEE estimation. The deuterium dose reaches equilibrium in urine between 3–5 hours, as observed in newborns [15], comparable with the study age group and has been standardised by the International Atomic Energy Agency [16,17]. Isotopic enrichments of ^2^H and ^18^O in urine samples were measured in duplicates using isotope ratio mass spectrometer (IRMS, Delta V advantage, Thermo Scientific, Bremen, Germany).

Total body water for body composition estimation was obtained by the deuterium dilution method and fat free mass (FFM) was derived using standardized protocols and calculations as per the International Atomic Energy Agency [16]. Similarly, established equations for rate of carbon dioxide production and energy expenditure were used for TEE estimation [16].

The participants were also assessed for the presence of impaired intestinal permeability with the dual sugar absorption test, using lactulose (L) and rhamnose (R) sugars (henceforth referred to as the LR protocol) in a short duration (2 h) LR protocol [18,19]. Mothers were requested to collect the early morning urine void into a sterile container, which served as a baseline sample for the experiment. Mothers were directed to provide breakfast to the child early in the morning. On arrival at the research facility and confirmation of a 2 h post-prandial period, the children were encouraged to void urine before a dose of 1.3 g lactulose and 0.3 g rhamnose (Tokyo Chemical Industry Co., Ltd) was administered orally in a volume of 14 mL water. Children were encouraged to drink water after dosing, but no other liquids and/or food was provided for the next 2 h. During this 2 h period all urine voids were collected through a pre-weighed plastic diaper lined with a sterile cotton pad. If no urine was collected at the end of 2 h, the collection was continued for an additional 0.5 h, in the fasting state. The diapers were weighed to quantify volume, the urine sample was transferred into a sterile container and stored in an ice box until the end of the experiment, when the samples were pooled in proportion to the total volume voided, and aliquoted into sterile cryovials and stored at -20⁰C until analysis.

Urinary L and R concentrations were measured by an isotope dilution method modified from a procedure that was previously described [20]. Briefly, a weighed volume of urine sample was spiked with a known quantity of U-^13^C_12_-lactulose and U-^13^C_6_-rhamnose:H_2_O (Cambridge Isotope Laboratories, MA, USA) as Internal Standards (IS). The samples were extracted and derivatized to their silylated esters by following two step procedure of methoxyamination and silylation by using methoxyamine hydrochloride, pyridine and *N*,*O*-bis(trimethylsilyl)trifluoroacetamide containing 1% trimethylchorosilane, Sigma-Aldrich MO, USA) and were analysed by gas chromatography-mass spectrometry (GC-MS, SQ, 5975, Agilent Technologies, CA, USA) in selected ion monitoring mode to quantify ions at *m/z* 361 and 367 (for lactulose and ^13^C_12_-lactulose respectively) and *m/z* 117 and 119 (for rhamnose and ^13^C_6_-rhamnose respectively).

Calculation of percent recovery of each sugar in the urine and their ratio was as follows:

Percent (%) lactulose (or rhamnose) recovery = [(total urine volume (mL) over first 2 h x lactulose (or rhamnose) concentration (μmol/L) in urine)/dose of lactulose (or rhamnose) administered] x 100

Lactulose to rhamnose ratio (LRR) = % lactulose recovery/% rhamnose recovery

The participants were categorized as having normal or high intestinal permeability (and associated inflammation) using an LRR value of 0.067 as the upper limit of normal [21]. This upper limit of normal (98^th^ centile, mean + 2SD) for LRR was obtained from 20 healthy children (LRR mean ± SD: 0.029 ± 0.019) with normal nutritional indices (LAZ, WAZ, and WHZ > -1) belonging to a high socio-economic background, recruited from the well-baby clinic of St. John’s Medical College and Hospital, Bangalore, for the larger study from which participants for the current study were selected (manuscript under preparation).

### Collection of fecal samples

Fecal samples were collected by the participants’ mothers in their respective households. Prior to sample collection, the field workers had demonstrated how to collect the fecal samples to the mothers, and clarified any doubts or questions that the mothers had regarding the sample collection procedure. A fecal sample collection kit, containing a sterile container, plastic bags, gloves and spoon was provided to the mothers to collect the first passage of faeces, in the morning hours, or according to the child’s routine. With gloved hands, the mothers used the spoon to transfer the fecal material from the plastic bags, on which the child had passed stool, to the sterile container. The collected fecal samples were either stored by the parents in the children’s homes at room temperature for a maximum of 5 hours, or collected directly from the children when feasible, before they were transferred in an ice box (2 to 8^°^C) to St. John’s research facility by the field workers, and then aliquoted into 3 sterile cryovials and stored at -80°C freezer until analysis. The fecal sample collection protocol was designed based on earlier studies that have demonstrated stability of fecal samples and associated microbiota profiles when collected and stored at 4°C or room temperature up to 24 hours before longer-term storage at -80°C till analysis [22,23].

### Fecal microbiota analysis

Fecal DNA was extracted using an in-house kit that comprised of fecal sample lysis, RNase A treatment, phenol-chloroform-isoamyl alcohol-based phase separation and precipitation of DNA with 100% ethanol. The extracted DNA was quantified using Qubit dsDNA HS Assay kit (Thermo Fisher Scientific, MA, USA) and checked on agarose gel for integrity. The extracted DNA was diluted to 5ng and V3-V4 amplicons were generated from 16S rDNA PCR products with region-specific primers (16S Forward: AGAGTTTGATCCTGGCTCAG; 16S Reverse: GGTTACCTTGTTACGACTT; V3-V4 Forward: CCTACGGGNGGCWGCAG; V3-V4 Reverse: GACTACHVGGGTATCTAATCC) and NEBNext High-Fidelity 2X PCR Master Mix (New England Biolabs, MA, USA) using a nested PCR strategy. The V3-V4 amplicons were then checked on agarose gel followed by clean up using AMPure XP beads (Beckman Coulter Inc., CA, USA).

Cleaned V3-V4 amplicons were used for library preparation using NEBNext Ultra DNA Library preparation kit (New England Biolabs, MA, USA). In brief, the amplicons were end repaired and mono-adenylated at 3’-end in a single enzymatic reaction. Next, NEB hairpin-loop adapters were ligated to the DNA fragments in a T4-DNA ligase-based reaction. Following ligation, the loop containing Uracil was linearized using USER Enzyme (a combination of Uracil-DNA glycosylase and Endonuclease VIII), to make it available as a substrate for PCR based indexing in the next step. During PCR, barcodes were incorporated using unique primers for each of the samples by giving 10 PCR cycles, thereby enabling multiplexing.

The prepared library was checked for fragment distribution using Agilent D1000 Screen Tapes and reagents (Agilent Technologies, Inc., CA, USA). The obtained library was pooled and diluted to final optimal loading concentration before Cluster amplification on Illumina flow cell. Once the cluster generation is completed, the clustered flow cell is loaded on Illumina HiSeq2500 instrument (Illumina, Inc., CA, USA) to generate 0.5M, 250bp Paired end reads/sample. To segregate the barcodes sequenced together on the machine, Illumina’s bcl2fastq v2.18 was used for demultiplexing. The default parameters of bcl2fastq were retained for the demultiplexing step which notably includes allowing one mismatch in the barcode sequence. The data quality post-demultiplexing was verified by custom scripts and found to be suitable for further analysis. These analyses were performed at MedGenome Labs Pvt Ltd, Bangalore, India.

### Bioinformatics and statistical analysis

#### Quality control and pre-processing of metagenomic sequences

The demultiplexed sequencing reads were processed using the Quantitative Insights Into Microbial Ecology QIIME 2 (v2019.10.0) pipeline [24]. The paired end reads were depleted of amplicon primers using the Cutadapt plugin [25]. Quality check was performed using demux plugin followed by denoising, chimera identification and PhiX removal and dereplication using the DADA2 plugin [26]. The final read statistics after the pre-processing steps are described in S1 Table. The minimum final read count after post-feature filtering was 60623 reads.

#### Taxonomy classification

Taxonomies were assigned using a naïve Bayes classifier (q2-feature- classifier and classify-sklearn plugins) trained on V3-V4 sequence region extracted from the latest SILVA rRNA (16S SSU) v132 reference database [27] using locus-specific primer sequences. Operational taxonomic units (OTUs) were defined at 99% similarity.

#### Phylogenetic diversity analysis

De novo sequence alignments were performed using MAFFT [28] followed by construction of the phylogenetic tree using FastTree [29]. OTUs present in less than 2 samples or at total counts below 10 were filtered out to remove potential PCR or sequencing errors and low abundant features that may not represent the true biological diversity. Alpha rarefaction plots indicated the completeness of OTU representation across different sequence sampling depths (S1 Fig). Measures of within-sample and between-sample phylogenetic richness and community consistency were evaluated by estimating alpha and beta diversity indices using rarefied data subsampled to minimal depth (60623 reads). The effect of selected sampling depth on alpha diversity indices (Pielou’s evenness, Faith’s phylogenetic diversity index, Shannon diversity and observed OTUs) (S2 Fig) and beta diversity indices (Bray-Curtis, Jaccard’s, unweighted and weighted UniFrac distances) were determined (S3 Fig). Principal coordinates analysis (PCoA) plots for the beta diversity metrics were generated using Emperor [30]. Group significances in the alpha and beta diversity metrics were assessed with Kruskal–Wallis test and permutational multivariate analysis of variance (PERMANOVA, at permutations = 999) respectively using the QIIME2 plugins.

#### Covariate analysis

The association of demographic, anthropometric and other variables with PCoA ordination (calculated based on Bray-Curtis dissimilarity) was estimated with envfit function in the vegan R package (999 permutations, followed by Benjamini-Hochberg multiple test correction) that utilizes MANOVA and linear correlations for categorical and continuous variables, respectively. Partial least squares discriminant analysis (PLS-DA) was performed using mixOmics R package using the case and non-case definitions for stunting, wasting and underweight groupings.

#### Univariate association analysis

Towards removing rare OTUs and improving the signal-to-noise ratio, only OTUs with relative abundance > 0.1% in at least 2 samples [31,32] were retained for this feature-based analysis. The final OTU table used for all subsequent analyses contains a total of 142 taxa including few taxonomic assignments at the subspecies level. Given the limited accuracy of using 16s rRNA (V3-V4) sequences for subspecies-level assignments, it is imperative to validate all reported associations at the subspecies level using targeted PCR-based approaches. The associations with anthropometric, demographic body composition and intestinal permeability parameters were assessed using Spearman correlation analysis on relative abundance measures. The p-values were adjusted for multiple comparison at each taxonomic level separately using Benjamini-Hochberg method. The association tests were performed using R platform [33].

#### Metagenomic pathway analysis

Metagenomic inference analysis was conducted by predicting KEGG orthology (KO) metagenomes [34], enzyme commission (EC) metagenomes and MetaCyc pathways [35] using PICRUSt (phylogenetic Investigation of Communities by Reconstruction of Unobserved States) [36] through q2-picrust2 plugin from QIIME2.

#### Linear discriminant analysis (LDA) Effect Size (LEfSe) analysis

The final filtered OTU table was analysed using the linear discriminant analysis (LDA) Effect Size (LEfSe) method [37] to compare case and non-case classes identified for stunting, wasting and under-nutrition groups as well as for the high and low LRR groups using default parameters. Relative abundances of the different KEGG pathways, EC terms and MetaCyc pathways were also assessed using LEfSe algorithm after placing a filter of 0.1% in at least two samples.

## Results

### Participant characteristics

Fecal samples from 41 participants (aged 18–24 months) were used for 16S rRNAV3-V4 sequencing based gut microbiota analysis. Participant characteristics are available in Table 1, categorized based on their nutritional status as stunted n = 16, non-stunted n = 25; wasted n = 8, non-wasted n = 33; underweight n = 15, normal weight n = 26 and in S2 Table, categorized based on their lactulose rhamnose ratio (LRR, ≥0.067: n = 16 and <0.067: n = 25). Based on their paediatric clinical examination at the time of recruitment and medical history obtained through caregiver interviews, the participants were otherwise normal, without renal affection, either due to disease or drugs.

**Table 1:** Characteristics of children categorised as cases and non-cases by nutritional status*.

	Stunted (LAZ<-2)	Non-stunted (LAZ≥-2)	P-value	Underweight (WAZ<-2)	Non-Underweight (WAZ≥-2)	P-value	Wasted (WLZ<-2)	Non-Wasted (WLZ≥-2)	P-value
n	16	25		15	26		8	33	
Sex (F/M)	8/8	10/15		9/6	9/17		5/3	13/20	
Age (months)	21.9 ± 2.6	21.2 ± 2.6	0.393	21.9 ± 2.7	21.3 ± 2.6	0.450	22.0 ± 3.3	21.4 ± 2.5	0.553
Weight (kg)	8.7 ± 0.8	9.9 ± 1.1	0.001	8.4 ± 0.6	10.0± 0.9	<0.001	8.4 ± 0.8	9.7± 1.0	0.002
Height (cm)	77.4 ± 2.4	81.0 ± 3.2	<0.001	77.7 ± 3.1	80.7 ± 3.1	0.004	79.5 ± 3.9	79.7 ± 3.3	0.910
Maternal education (years)	8 (5,10)	10 (7,10)	(0.095)	8 (3,10)	9 (7,10)	(0.369)	6 (4,10)	10 (7,10)	(0.165)
Hb (g/dL)	10.2 (8.9,11.1)	10.6 (8.8,11.1)	(0.500)	10.8 (10.4,11.4)	10.1 (8.6,10.8)	(0.086)	11.0 (10.6,11.4)	10.3 (8.7,10.9)	(0.112)
%FFM^^^	77 (74,82)	78 (75,82)	(0.805)	77 (74,81)	78 (74,82)	(0.779)	77 (75,82)	78 (74,82)	(0.957)
TEE (kcal/d)^^^	783 ± 160	768 ± 220	0.837	711 ± 87	807 ± 224	0.203	648 ± 99	800 ± 198	0.108
TEE (kcal/kg FFM/d)^^^	116 ± 20	99 ± 24	0.050	111 ± 13	104 ± 27	0.517	102 ± 18	108 ± 24	0.605
LAZ	-2.5 ± 0.4	-1.1 ± 0.8	<0.001	-2.3 ± 0.6	-1.2 ± 0.9	<0.001	-1.6 ± 1.3	-1.6 ± 0.9	0.961
WAZ	-2.3 ± 0.6	-1.2 ± 0.8	<0.001	-2.5 ± 0.4	-1.1 ± 0.6	<0.001	-2.6 ± 0.6	-1.4 ± 0.8	<0.001
WLZ	-1.4 ± 0.8	-0.9 ± 1.0	0.069	-1.9 ± 0.6	-0.6 ± 0.8	<0.001	-2.4 ± 0.3	-0.8 ± 0.8	<0.001
**Dual sugar assay**									
Rhamnose recovery (%)	1.5 (1.2,1.9)	1.6 (1.2,2.1)	(0.741)	1.6 (1.3,2.1)	1.5 (1.2,2.0)	(0.678)	1.8 (1.2,2.7)	1.5 (1.2,1.9)	(0.507)
Lactulose recovery (%)	0.08 (0.06,0.15)	0.09 (0.05,0.19)	(0.843)	0.14 (0.06,0.21)	0.07 (0.05,0.13)	(0.301)	0.14 (0.07,0.21)	0.07 (0.05,0.16)	(0.235)
LRR	0.052 (0.042,0.094)	0.059 (0.039,0.090)	(0.947)	0.052 (0.045,0.138)	0.057 (0.034,0.087)	(0.512)	0.052 (0.050,0.103)	0.056 (0.033,0.090)	(0.467)

*Values are mean ± SD and Median (Q1,Q3), P value for Independent t-test (Mann-Whitney test).

^^^n = 13 and 17 for stunted and non-stunted, 10 and 20 for underweight and non-underweight, 5 and 25 for wasted and non-wasted.

Abbreviation: LAZ, Length-for-age z-score; WAZ, Weight-for-age z-score; WLZ, Weight-for-Length z-score; Hb, Haemoglobin; FFM, fat free mass; %FFM, % fat free mass per kilogram body weight; TEE, Total Energy Expenditure LRR, Lactulose Rhamnose ratio.

### 16S rRNA V3-V4 sequencing of fecal microbiota

A total of 47,363,558 read sequences were obtained across 41 samples, with an average of 11,55,208 reads per sample (S1 Table). After preprocessing using QIIME2 pipeline, 6,429,231 good quality reads were obtained with an average of 156,810 reads per sample. The detected operational taxonomic unit (OTU) counts ranged between 158 and 534.

### Fecal microbiota is similarly diverse in nutritional indicator groups

Alpha rarefaction curves were generated to assess the effect of different sampling depth on OTU abundance (S1A Fig). The plots clearly indicate that detection of observed OTUs had already attained a plateau at 60000 reads, the minimum sample depth in the dataset. The plateauing trend was separately confirmed for the case and non-case groupings of the three nutritional status groups as well (S1B–S1D Fig). Several alpha and beta diversity estimators were computed to measure within-sample and between-sample diversities and to compare cases and non-cases within the three nutritional status groups. Overall, alpha diversity was not different between case and non-case groups (S2 Fig) except in case of Shannon diversity measures among underweight children (p = 0.048). However, a few of the beta diversity metrics were different between cases and non-cases, such as Bray-curtis distance in wasted (p = 0.01) and weighted UniFrac distances in stunted (p = 0.04) and wasted (p = 0.006) children (S3 Fig). Overall, there was a significant overlap between cases and non-cases using principal coordinates analysis of beta diversity measures.

### Child weight and weight-based scores are covariates of fecal microbiota variation, specifically in boys

**Fig 1:**
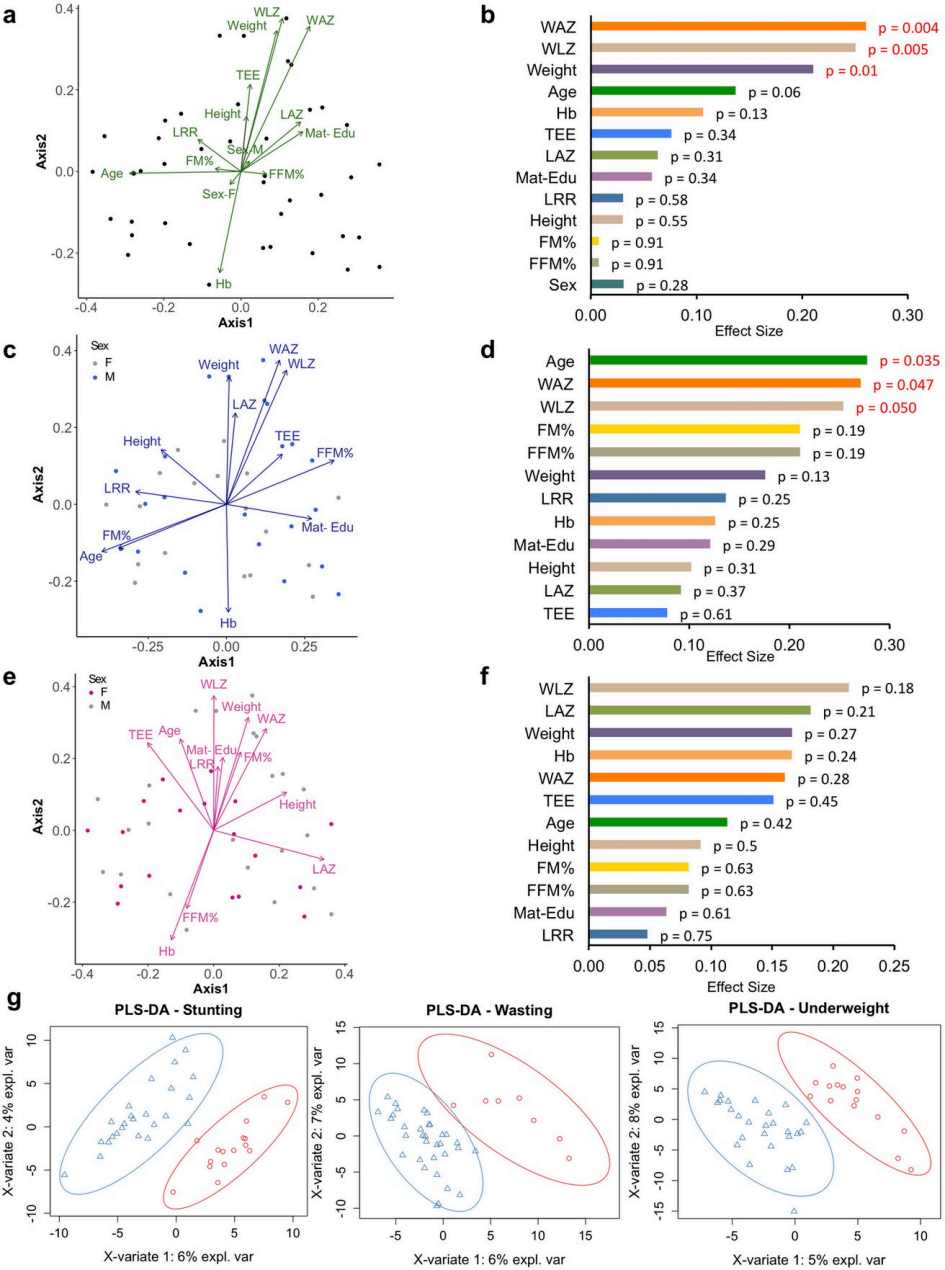
Microbial community variation among 18–24 month old Indian children. Microbial community variation represented by Principal coordinates analysis (PCoA) based on Bray-Curtis distances highlighting the covariate contribution to community variation estimated using envfit correlation analysis plotted against first two components with arrows scaled to contribution in (A) all 41 participants, (C) boys (n = 23) and (E) girls (n = 18). Effect sizes of covariates of microbial composition in (B) all 41 participants, (D) boys (n = 23) and (F) girls (n = 18), significance indicated with p-values (p < 0.05). (G) PLS-DA score plot showing model discrimination between cases and non-cases with respect to stunting, wasting and underweight status, respectively. The ellipses denote 95% confidence intervals for each grouping. LAZ: length-for-age Z-score, WAZ: weight-for-age Z-score, WLZ: weight-for-length Z-score, TEE: total energy expenditure, LRR: lactulose to rhamnose ratio, FM: fat mass, FFM: fat free mass.

Towards identifying covariates of microbiota variation in our sample set, we performed Principal Coordinates Analysis (PCoA) based on their Bray-Curtis dissimilarity measures and evaluated possible correlations of the microbial community structure with anthropometric, demographic, body composition, TEE and intestinal permeability parameters (Fig 1A). Weight and weight-derived scores (WAZ, WLZ) had significant effect on sample clustering (Fig 1B). The maximum individual effect size estimated was for WAZ (26%), followed by WLZ (25%) and weight (21%). Subgroup analysis of the covariate effects by sex revealed that the weight-mediated effects were specifically present in the boys (Fig 1C–1F). A significant contribution of age was also observed within the boys (p = 0.035). In contrast, none of the covariates tested had any significant effect on fecal microbial community variation in the girls. Further examination of the taxonomic relative abundances using supervised PLS-DA analysis revealed strong separation between the case and non-case samples within the three nutritional status categories, with maximum separation observed for underweight category (Fig 1G).

### Bacterial abundance associates with growth measures and age

To delineate relations between anthropometric, demographic, body composition, TEE and intestinal permeability variables and nutritional status with microbial abundances, the association of age, LAZ, WAZ, WLZ, %FFM, %FM, TEE, LRR and Hb levels of the children was tested with the relative abundances, using Spearman correlation analysis (Table 2, S4 Fig). An abundance-filtered OTU table consisting of 142 taxa was used for all downstream feature-based analysis in an attempt to improve the signal-to-noise ratio by additionally filtering out rare OTUs. WAZ was negatively associated with Bacteriodetes Phylum (p.adj = 0.01), as well as its related class (Bacteroidia, p.adj = 0.02) and order (Bacteroidales, p.adj = 0.03). WLZ was also negatively associated with Bacteriodetes Phylum (p.adj = 0.03) and its class (Bacteroidia, p.adj = 0.05). At the phylum level, a positive association was identified between WLZ and Firmicutes abundance (p.adj = 0.03). The abundances of Coriobacteria and Erysipelotrichia classes showed positive associations with WAZ (p.adj = 0.04) and WLZ (p.adj = 0.04), respectively. Hemoglobin levels were found to be negatively correlated with *Enterobacter* abundance (p.adj = 5.3e-03) at the genus level. Finally, at the species level, significant positive associations were identified between *Bifidobacterium longum subsp longum* abundance and WAZ (p.adj = 0.02). Significant negative associations were observed between age and abundances of *Bifidobacterium bifidum* (p.adj = 0.009) and *Bifidobacterium breve* (p.adj = 0.03). No statistically significant associations were identified for sex, LAZ score, %FFM, %FM, TEE and LRR (S3 Table).

**Table 2:** Association of fecal microbiota with nutritional status indicated by length-for-age (LAZ), weight-for-age (WAZ) and weight-for-length (WLZ) Z-scores determined by Spearman correlation analysis.

Taxon	Variable	Spearman		
		ρ	p	p.adj
**Phylum**				
Bacteroidetes	WAZ	-0.46	0.002	0.013
Bacteroidetes	WLZ	-0.39	0.011	0.032
Firmicutes	WLZ	0.40	0.010	0.032
**Class**				
Bacteroidia	WAZ	-0.46	0.002	0.022
Bacteroidia	WLZ	-0.39	0.011	0.053
Coriobacteriia	WAZ	0.41	0.008	0.040
Erysipelotrichia	WLZ	0.44	0.004	0.043
**Order**				
Bacteroidales	WAZ	-0.46	0.002	0.029
**Genus**				
*Enterobacter*	Hb	-0.57	9.6E-05	5.3E-03
**Species**				
*Bifidobacterium longum subsp longum*	WAZ	0.52	0.0005	0.017
*Bifidobacterium bifidum*	Age	-0.54	0.0003	0.009
*Bifidobacterium breve*	Age	-0.47	0.002	0.033

Abbreviation: WAZ, Weight-for-age z-score; WLZ, Weight-for-Length z-score; Hb, Haemoglobin.

### Nutritional status associated Taxonomic composition of fecal samples

**Fig 2:**
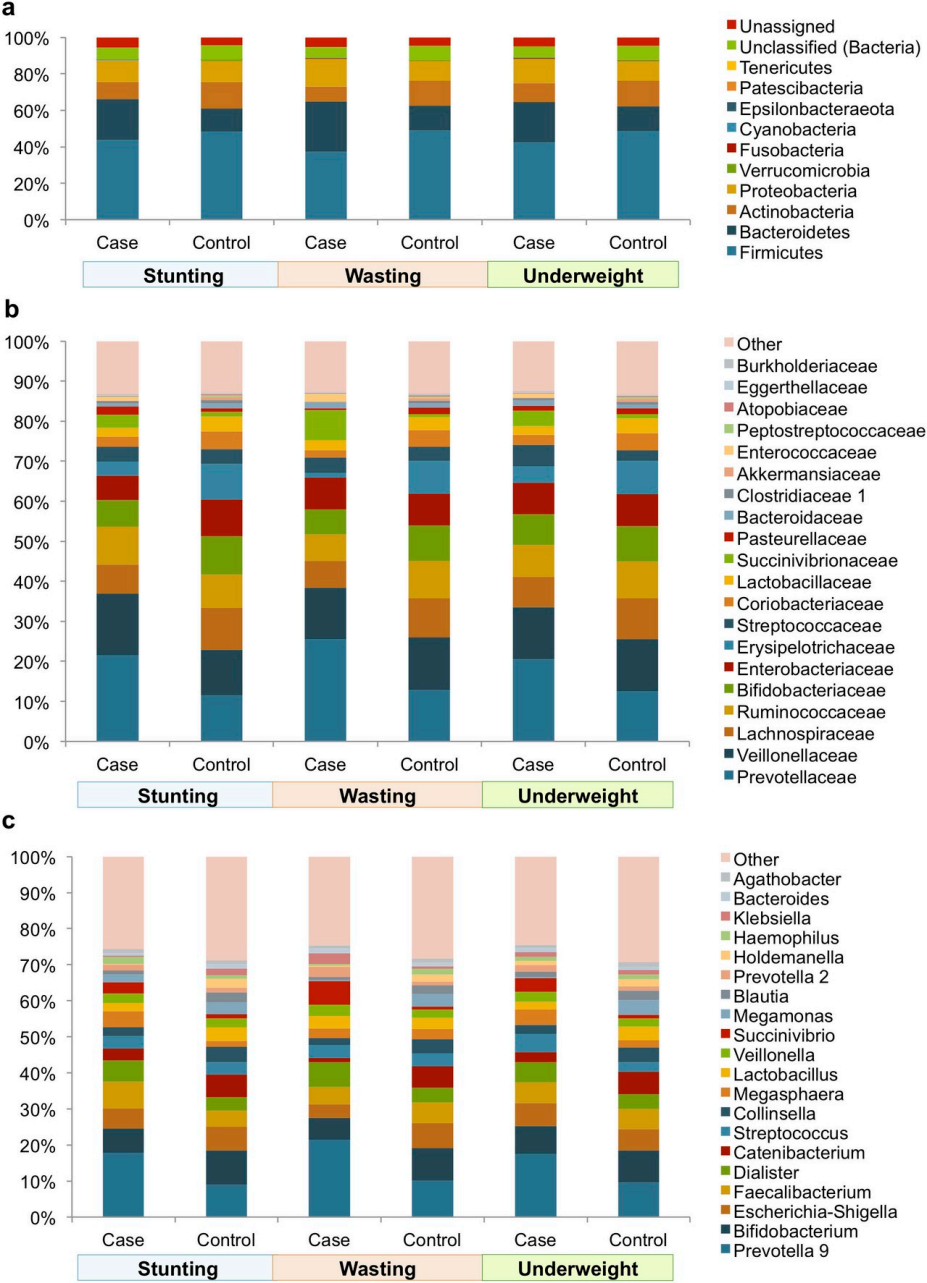
The fecal microbial composition profiles of 18–24 month old Indian children. Fecal microbial composition profiles at the (**A**) phylum level and (**B**) family level and (**C**) genus level within the case and non-case samples of stunting, wasting and underweight groupings. Only the top 20 families with most relative abundance are labelled in (B) and (C). The remaining families and genera including unclassified and unassigned have been grouped together as “Other”.

To discern the overall microbial composition profile of the three nutritional status groups, taxonomic classification of the sequencing reads originating from each sample using the SILVA database were performed and their relative proportions visualized. Ten bacterial phyla were detected in the fecal samples with Firmicutes being the most prevalent (44.7%), followed by Bacteriodetes (18.9%), Actinobacteria (11.7%) and Proteobacteria (12.2%) (Fig 2A). Among the 61 bacterial families identified, the most abundant were Prevotellaceae (17.6%), Veillonellaceae (13.2%), Lachnospiraceae (8.6%), Ruminococcaceae (8.5%) and Bifidobacteriaceae (8%) (Fig 2B). At the genus level, the fecal microbiota was dominated by *Prevotella 9* (14.4%), *Bifidobacterium* (8%), *Escherichia-Shigella* (5.8%), *Faecalibacterium* (5.6%) and *Dialister* (5.1%). A total of 12 genera including *Bifidobacterium*, *Collinsella*, *Streptococcus*, *Veillonella*, *Faecalibacterium*, *Dialister*, *Escherichia-Shigella*, *Prevotella 9*, *Brevibacillus*, *Blautia*, *Dorea* and *Butyricicoccus* were detected in at least 95% of all samples (39/41) (Fig 2C). Visual inspection of the taxonomic distribution suggested distinct case-non-case differences that were further subjected to statistical tests using the linear discriminant analysis Effect Size (LEfSe) algorithm (see below).

### Differential microbial abundance with nutritional status

**Fig 3:**
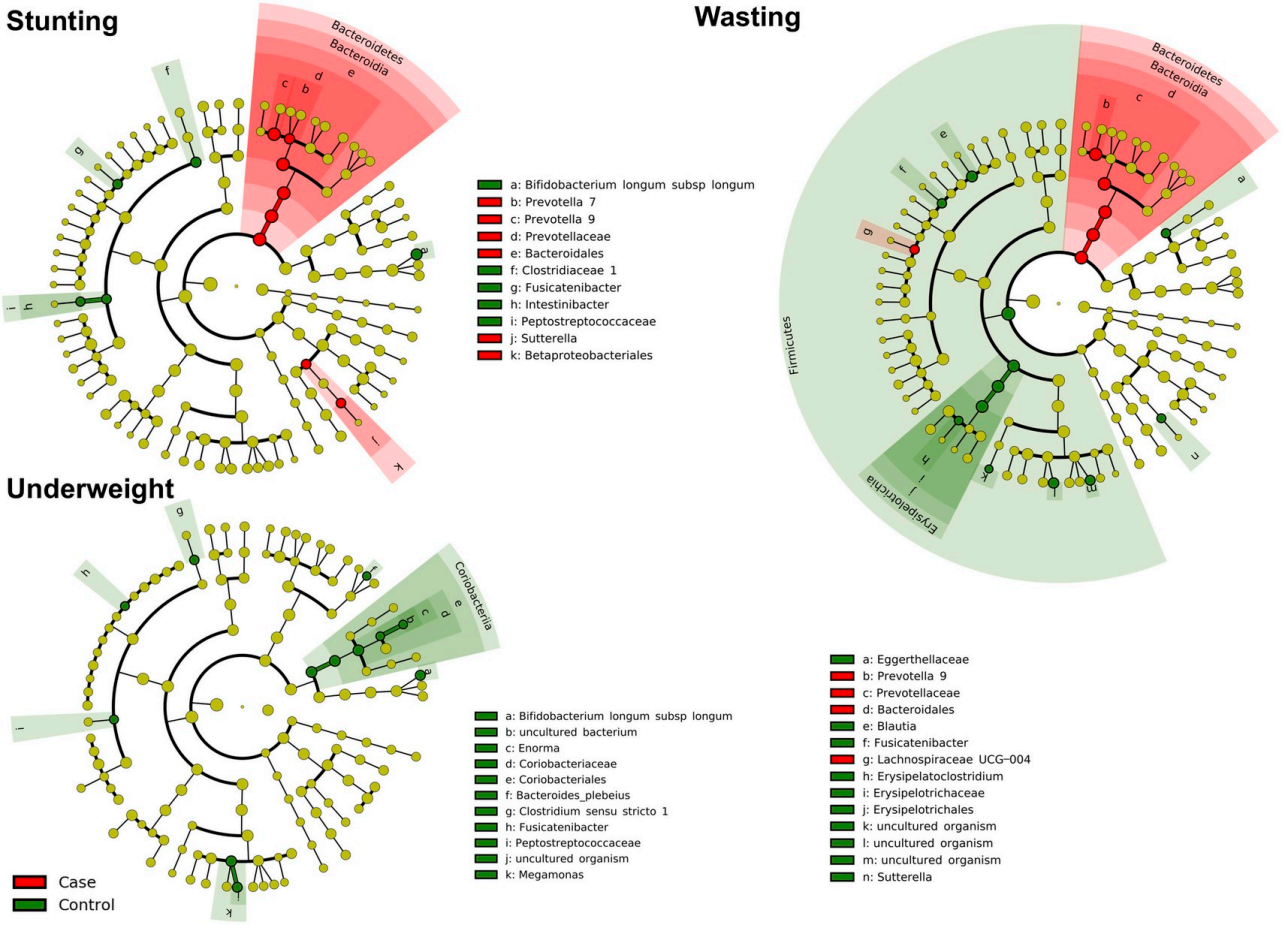
Fecal microbiota characterization cladograms of stunting, wasting and underweight in 18–24 month old Indian children. The fecal microbiota characterization cladograms were based on LEfSe (linear discriminant analysis Effect Size) in the study participants.

To identify differences in bacterial taxon abundances, statistical comparisons were made in the three nutritional status categories using the linear discriminant analysis Effect Size (LEfSe) algorithm. Stunted children showed enrichment for bacterial genera including *Prevotella 7* and *Prevotella 9* of Prevotellaceae family and *Sutterella* of Betaproteobacteriales order. They also showed depletion of Clostridiaceae 1 family, *Intestinibacter* and *Fusicatenibacter* genera and *Bifidobacterium longum subsp longum* species compared to non-stunted children (Fig 3, S4 Table). *Prevotella 9* of Prevotellaceae, *Lachnospiraceae UCG-004* genus and Bacteroidales order were found over-represented in wasted children accompanied by decreased abundance of *Blautia*, *Fusicatenibacter*, *Sutterella* and *Erysipelatoclostridium* genera, Eggerthellaceae family and uncultured species within *Megasphaera*, *Megamonas* and *Phascolarctobacterium* genera. The comparative analysis also highlighted significant reduction of uncultured species belonging to *Enorma* and *Megamonas* genera, *Bifidobacterium longum subsp longum* species, *Clostridium sensu stricto 1* and *Fusicatenibacter* genera, along with Peptostreptococcaceae family among underweight children.

### Predicted microbial function differences in malnourished children: Lipopolysaccharide biosynthesis pathway is upregulated in stunted and wasted children

To investigate whether alterations in microbial metabolic functions correlate with malnutrition indices, PICRUSt was employed to infer functional gene content associated with taxonomic composition. Further, differential abundance of PICRUSt-predicted KEGG and MetaCyc pathways and enzymes in the nutritional status categories were assessed using LEFSe algorithm. Owing to limited sample size, a linear discriminant analysis (LDA) cut-off score of 2 or greater and an unadjusted p-value threshold of 0.05 were used to report differentially abundant functional entities identified.

**Fig 4:**
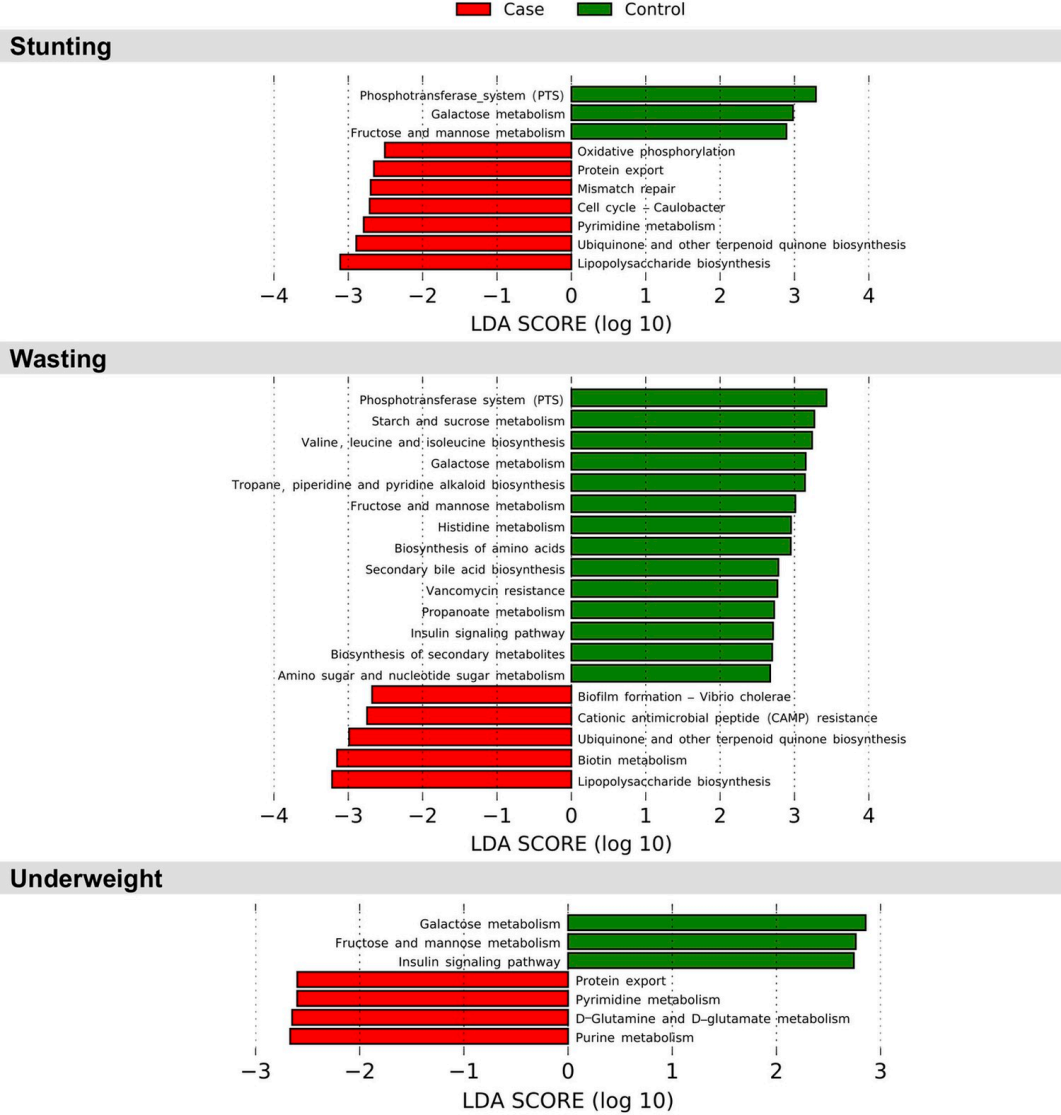
Predicted microbial pathways (KEGG) of stunting, wasting and underweight in 18–24 month old Indian children. A summary of differential abundant microbial pathways (KEGG) predicted in stunted, wasted and undernourished children using the linear discriminant analysis (LDA) Effect Size (LEfSe) tool. An LDA cut-off score of 2 or greater and unadjusted p-value threshold of 0.05 was used to report the significant findings.

Out of the 111 KEGG pathways tested, a total of 10, 19 and 7 pathways were found to be significantly associated with stunting, wasting and underweight status, respectively (Fig 4, S5A Table). Lipopolysaccharide biosynthesis and ubiquinone and terpenoid quinone synthesis pathways were upregulated in case fecal samples of stunting and wasting categories.

### Macronutrient metabolism pathways in fecal microbiota from undernourished children are perturbed

KEGG pathways involved in galactose, fructose and mannose metabolism were uniformly depleted in case fecal samples of stunting, wasting and underweight categories. The phosphotransferase system pathway was depleted in both stunted and wasted case fecal samples but not underweight ones. Protein export and nucleotide metabolism pathways like pyrimidine metabolism were upregulated in case fecal samples of stunting and underweight categories.

LEfSe Analysis of EC terms also highlighted similar findings (S5 Fig, S5B Table). Pyrimidine metabolism enzyme, thymidine kinase and protein export enzyme, signal peptidase 1 were found to be enriched in case fecal samples belonging to every nutritional status grouping. Stunted and underweight children also showed increased abundance of starch synthase (glycosyl-transferring) enzyme. An aminoacyl-tRNA biosynthesis enzyme, cysteine-tRNA ligase was found to be under-represented among the cases of all three nutritional status categories. In addition, phosphatidylglycerol-membrane-oligosaccharide glycerophosphotransferase, an enzyme involved in glycerolipid metabolism, showed reduced abundance in stunted and wasted children. Wasted and underweight children showed depletion of fructokinase and 4-hydroxy-3-methylbut-2-en-1-yl diphosphate reductase.

In the MetaCyc pathway analysis, Kdo transfer to lipid IV_A_ III (Chlamydia), (5Z)-dodecenoate biosynthesis I and ADP-L-glycero-beta-D-manno-heptose biosynthesis pathways were over-represented among cases of all the three groupings (S5C Table). A total of 16 pathways were jointly perturbed in stunted and wasted children including depletion of predicted amino acid biosynthesis pathways such as arginine via N-acetyl-L-citrulline, arginine via ornithine and isoleucine biosynthesis II along with enrichment of an array of fatty acid biosynthesis sub-pathways among others (S6 Fig). Sucrose degradation III, pentose phosphate pathway and glycogen biosynthesis I pathways were depleted among wasted and underweight children while tetrahydrofolate biosynthesis superpathway and 4-deoxy-L-theo-hex-4-enapyranuronate degradation pathways were highly abundant in the cases belong to both groups. Both stunted and underweight fecal samples recorded remarkable abundance of inosine 5’-phosphate degradation pathway.

### Differential microbial abundance and functions with intestinal permeability

**Fig 5:**
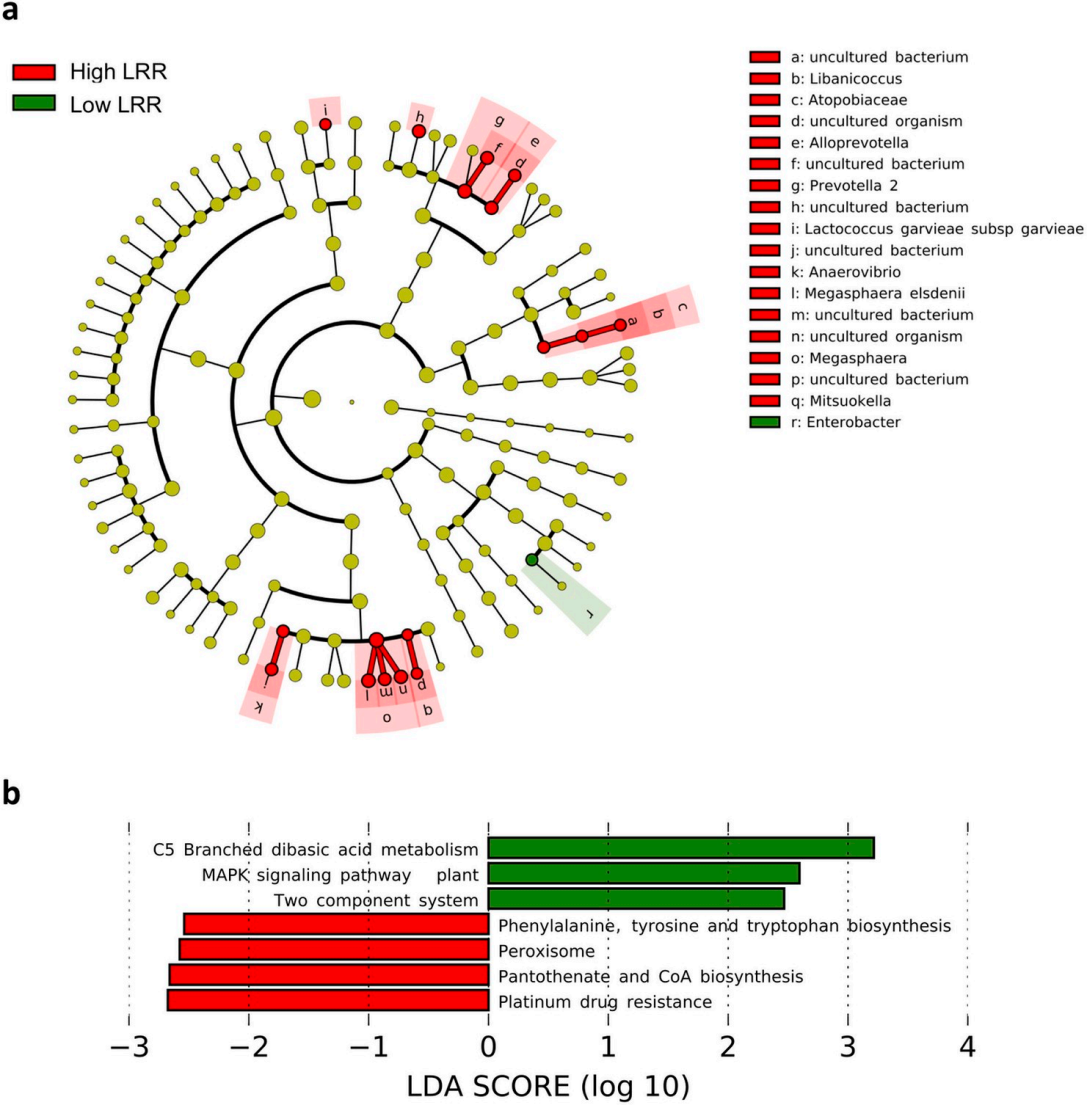
Fecal microbiota characterization based on lactulose-rhamnose ratios in 18–24 month old Indian children. **(A)** Fecal microbiota characterization cladograms of high LRR and low LRR groups based on LEfSe (linear discriminant analysis Effect Size) in the study participants. **(B)** Differentially abundant microbial pathways (KEGG) predicted in high LRR and low LRR groups using LEfSe analysis. An LDA cut-off score of 2 or greater and unadjusted p-value threshold of 0.05 was used to report the significant findings.

To identify gut microbial correlates of intestinal permeability, we applied the LefSe method to compare the microbial levels in individuals categorized into high LRR (LRR ≥ 0.067) and low LRR (LRR < 0.067) groups. The relative abundances of *Megasphaera elsedenii*, *Megasphaera*, *Mitsuokella*, *Lactococcus garvieae subsp garvieae*, *Prevotella 2*, *Alloprevotella*, *Anaerovibrio and Libanicoccus* were significantly higher for the high LRR group, while the relative abundances of Enterobacter was reduced compared to the low LRR group with LDA scores over 2 and unadjusted p-value < 0.05 (Fig 5, S6 Table).

Comparison of predicted microbial functions of the two groups suggested that the high LRR group is likely to have reduced capacity for microbial functions associated with C5-Branched dibasic acid metabolism and environmental information processing pathways such as Two component system and MAPK signalling pathway–plant. Pathways associated with platinum drug resistance, pantothenate and CoA biosynthesis, peroxisome and phenylalanine, tyrosine and tryptophan biosynthesis were predicted to be enriched among the high LRR group.

Similar analysis involving MetaCyc pathways revealed upregulation of partial TCA cycle (obligate autotrophs) and biosynthesis of methionine and tetrahydrofolate in the high LRR group along with downregulation of urea cycle, L-rhamnose degradation I and dTDP-N-Acetylthomosamine biosynthesis (S7A Fig). In addition, two enzymes, (S)-2-haloaciddehalogenase and histidine-tRNA ligase were found to be overabundant among the high LRR group (S7B Fig, S6 Table).

## Discussion

The present analysis reports associations of the fecal microbiota with anthropometric and nutritional status metrics of stunting, wasting and underweight in a group of Indian children aged 18–24 months from urban slum dwelling families with low socioeconomic status. The underweight case samples had lower overall alpha diversity (Shannon index measure, for both richness and evenness) compared to the non-underweight. Other metrics of alpha diversity of the microbiota, evenness (Pielou’s evenness) and richness (Faith’s phylogenetic diversity index and observed OTUs) were similar in fecal samples from case and non-case groups for stunting, wasting and underweight. This finding is similar to the lower alpha diversity of the gut microbiota observed in older (9–11 year) stunted Mexican children [38], but is in contrast with the higher alpha diversity reported in stunted children aged 6–24 months from India in comparison to well-nourished ones [8]. Factors including diet diversity, quality of potable water, living conditions could explain these differences [39,40].

In the 41 fecal samples in this study, Firmicutes, Bacteroidetes and Actinobacteria were the top 3 abundant phyla and Prevotellaceae, Veillonellaceae and Lachnospiraceae were the top 3 abundant families. A longitudinal case study of a single, vaginally-born, healthy male infant from USA also reported Bacteroidetes and Firmicutes as the top 2 abundant phyla at 1.5–2 years age [41]. In partial similarity to our findings, another longitudinal study on 2 cohorts of >100 healthy Danish infants born either to mothers with normal weight or to obese mothers, reported Lachnospiraceae, Veillonellaceae and Prevotellaceae as the top most, 5^th^ and 12^th^ most abundant families in the fecal samples at 18 months of age [42]. *Prevotella 9*, *Bifidobacterium* and *Escherichia-Shigella* were the top 3 abundant genera in our study. This points to a likely *Prevotella* enterotype across nutritional categories in these children and is in line with the *Prevotella* enterotype [43], identified in 1–6 year old healthy children from rural Burkina Faso and in 7–11 year old Indonesian and Thai children reflecting their high intakes of resistant starch from carbohydrates and lower bile acid biosynthesis and associated with depleted fat intakes [44,45]. As *Prevotella* is known to degrade intestinal mucin glycoproteins [46], a *Prevotella*-enriched gut microbiome could potentially impair intestinal integrity. In line with this, Kristensen et al have reported *Prevotella* as the most and second-most abundant genus in fecal samples from 6–24 month old Ugandan children hospitalized for oedematous and non-oedematous severe acute malnutrition, respectively [47]. Further, unlike the healthy children from Burkina Faso [45] but like the stunted 2–5 year old children from Madagascar and the Central African Republic (CAR) [10], potentially enteropathogenic *Escherichia-Shigella* were the 3^rd^ highest abundant genera. Interestingly, *Streptococcus* and *Veillonella* that are normally found in the oropharyngeal cavity and were over-represented in fecal samples from the stunted children from Madagascar and CAR, were amongst the 12 genera present in ≥95% of our study samples and were 7^th^ and 11^th^ highest abundant genera in our study. *Veillonella* was the 6^th^ most abundant genera in fecal samples from a set of 10 normal birth weight and non-stunted and 10 low birth weight and persistently stunted Indian children [8]. In the same vein, a *Streptococcus* and a *Veillonella* species in the duodenal samples from 1.5 year old children with EE were negatively correlated with their LAZ scores [5].

Of the anthropometric, demographic, body composition and intestinal permeability parameters tested, only weight, WAZ and WLZ acted as covariates of microbiota variation in the 41 samples. Interestingly, these parameters as well as age, were observed to be statistically significant covariates specifically in samples from boys. Sex-specific associations have been reported earlier in boys between their BMI at 5–8 years and antibiotic use within the first year of life [48]. To the best of our knowledge, the current study is the first report of child sex-specific associations of nutritional indicators with the gut microbiota profile.

Evaluation of associations between fecal microbial abundances and anthropometric, demographic, body composition, TEE and intestinal permeability variables revealed only 3 significant associations at species level. Abundances of *Bifidobacterium longum subsp longum* was positively associated with WAZ while those of *Bifidobacterium bifidum* and *Bifidobacterium breve* were negatively associated with age. Both *B*. *bifidum* and *B*. *breve* are well-known infancy and early childhood associated bacterial taxa [49], with another study reporting decrease in abundance of *B*. *bifidum* and *B*. *breve* between 1 to 2 years in a cohort of 87 healthy Norwegian children [50]. These observations tally well with their predicted functions, in fermenting human milk oligosaccharides by *B*. *bifidum* through its lacto-N-biosidase and galacto-N-biosidase activity [51] and in starch degradation by *B*. *breve* that encodes the starch degrading enzyme amylopullulanase, during the introduction of complementary feeding [52]. *B*. *longum* abundance has been shown to increase with prebiotic (inulin based plant-derived fermentable oligosaccharide) supplementation and associated loss of WAZ and %FM in 7–12 year old overweight/obese Canadian children [53], which is in contrast to observations from this study. Apart from inulin, legumes are a major source of plant-derived fermentable oligosaccharide in the diet [54]. Since legumes form the most important source of protein in predominantly vegetarian, low socioeconomic status Indian families, positive association of *B*. *longum subsp longum* abundance with WAZ in our study could simply be a reflection of legume consumption by these children, relating to better WAZ scores.

Microbial metabolic pathways demonstrate significant stability despite high inter-individual variability in their taxonomic composition [55]. In this study, macronutrient metabolism pathways were found to be perturbed in fecal samples from undernourished children, which could either be causal in nature or merely effect of dietary and other exposures leading to their undernourished state. Future nutritional intervention studies are likely to better resolve the role of gut microbial function in childhood growth faltering.

The lipopolysaccharide (LPS) biosynthesis pathway was upregulated in case fecal samples of stunting and wasting categories while biosynthesis pathway of its precursor, ADP-L-glycero-beta-D-manno-heptose, was over-represented in case samples from all 3 nutritional status groupings. Elevation of plasma LPS levels, a direct marker of gut microbial translocation across a compromised gut barrier, has been reported in ~15 month old malnourished Zambian children [56]. Gut microbial LPS is also known to be a potent activator of innate immune signalling and can influence the susceptibility of children to allergies and autoimmunity [57]. Based on observations from the current study, it is plausible to argue that elevation in plasma LPS levels could be associated with an increased capacity of the gut microbiota to synthesize LPS, which is likely related to increased abundance of related Gram-negative bacterial species.

On fecal analysis of cases and non-cases by LRR categories (high and low LRR) relative abundance of *Megasphaera*, *Mitsuokella* and *Lactococcus garvieae* was noted to be significantly higher in the high LRR group. These results replicate findings from a Malawian cohort of children (with mean age of 20 months) who were similarly categorised by lactulose mannitol ratio (LMR) cut-offs, wherein *Megasphaera*, *Mitsuokella*, and *Sutterella* were found to be more prevalent in those with high LMR, possibly indicating an intestinal inflammatory response in these children [58].

The key strength of this study resides in the ability to have characterized the gut microbiota of a largely homogenous group of Indian children aged 18–24 months from a single, urban slum, for whom demographic, anthropometric, body composition, TEE and intestinal permeability data were available. Additionally, subject sex categorized analysis of the associations between gut microbiota profiles and anthropometric and nutritional indicators, were performed, which helps to understand if sex of the child is likely to act as a modifier of future interventions to improve gut health and in turn child linear growth. The primary weakness of this study lies in its small sample size, in light of which the findings need to be interpreted. Further, the fecal samples had been stored at room temperature for a maximum of 5 hours before transfer in an ice box at 2 to 8^°^C to the study site for aliquoting and storage at -80°C till analysis. Though stability of fecal samples and associated microbiota profiles when collected and stored at 4°C or room temperature up to 24 hours before longer-term storage at -80°C till analysis has been reported [22,23], few other studies have recommended storage of fecal samples up to 4 hours at room temperature if storing at 4°C or freezing at -80°C immediately after collection is not feasible [59,60]. Finally, whole genome shotgun sequencing, instead of the 16S rRNA sequencing of the fecal samples, would have provided a more comprehensive understanding of the gut microbial profile of these children, which was not performed due to feasibility issues.

To conclude, this study describes the relations between nutritional indicators and fecal microbiota profiles in a group of young Indian children from a low socioeconomic status. Key findings include associations of bacterial abundances with WAZ and age, which were specific to samples from boys. In fecal samples from children belonging to stunted, wasted or underweight categories, bacterial macronutrient metabolism pathways were perturbed while pathways related to lipopolysaccharide synthesis and its precursor were upregulated. Future larger validation studies in populations from India and other geographical settings will provide critical evidence required to effectively modulate the gut microbiome for improving linear growth in children.

## Supporting information

S1 Fig Alpha-rarefaction curves. **(A)** Alpha-rarefaction curves showing observed OTU counts for all samples and samples grouped by **(B)** stunting status, **(C)** wasting status and **(D)** underweight status at different sample sequencing depths for maximum depth set as 60623 reads with 10 iterations per depth.
(PDF)

S2 Fig Box plots of alpha diversity measures (Pielou’s evenness, Faith’s phylogenetic diversity index, Shannon diversity and observed OTUs) between cases and non-cases within stunting, wasting and undernutrition groupings. The outlier samples are highlighted in blue and yellow colors for cases and non-cases respectively.
(PDF)

S3 Fig Emperor PCoA plots showing the clustering of samples based on beta diversity measures (Bray-Curtis, Jaccard’s, unweighted and weighted UniFrac distances). Each dot represents a sample. P-values indicate group significances comparing cases and non-cases within stunting, wasting and undernutrition groupings using PERMANOVA. P<0.05 are represented in red font.
(PDF)

S4 Fig Scatter plots of association between fecal bacterial taxa and nutritional status of the study participants indicated by length-for-age (LAZ), weight-for-age (WAZ) and weight-for-length (WLZ) Z-scores and Hemoglobin and Age. Associations were determined using Spearman correlation (ρ). p = raw p-value, q = fdr-adjusted p-value.
(PDF)

S5 Fig A summary of differential abundant microbial enzymes (EC terms) predicted in stunted, wasted and undernourished children using the linear discriminant analysis (LDA) Effect Size (LEfSe) tool. An LDA cut-off score of 2 or greater and unadjusted p-value threshold of 0.05 was used to report the significant findings.
(PDF)

S6 Fig A summary of differential abundant microbial MetaCyc pathways predicted in stunted, wasted and undernourished children using the linear discriminant analysis (LDA) Effect Size (LEfSe) tool. An LDA cut-off score of 2 or greater and unadjusted p-value threshold of 0.05 was used to report the significant findings.
(PDF)

S7 Fig Differentially abundant microbial (A) MetaCyc pathways and (B) enzymes (EC terms) predicted in high LRR and low LRR groups using LEfSe analysis. An LDA cutoff score of 2 or greater and unadjusted p-value threshold of 0.05 was used to report the significant findings.
(PDF)

S1 Table Data preprocessing statistics showing input raw read counts, final read counts after pre-processing and observed operational taxonomic units (OTUs) for each subject. (PDF)

S2 Table Characteristics of children categorised as cases and non-cases by nutritional status and lactulose rhamnose ratio. (PDF)

S3 Table Association of fecal microbiota with nutritional status indicated by length-for-age (LAZ), weight-for-age (WAZ), weight-for-length (WLZ) Z-scores, age lactulose rhamnose ratio (LRR), %fat free mass (FFM_perc), %fat mass (FM_perc), total energy expenditure (TEE) and haemoglobin (Hb) determined by Spearman correlation analysis. (XLSX)

S4 Table LEfSe (linear discriminant analysis Effect Size) analysis of gut microbiome in the study participants based on Stunting, Wasting and Underweight. (XLSX)

S5 Table Differential abundance analysis of microbial KEGG pathways, enzymes and MetaCyc pathways. **(A)** Differentially abundant microbial pathways (KEGG) predicted in stunted, wasted and undernourished children using the linear discriminant analysis (LDA) Effect Size (LEfSe) tool. **(B)** Differentially abundant microbial enzymes (EC terms) predicted in stunted, wasted and undernourished children using the linear discriminant analysis (LDA) Effect Size (LEfSe) tool. **(C)** Differentially abundant microbial MetaCyc pathways predicted in stunted, wasted and undernourished children using the linear discriminant analysis (LDA) Effect Size (LEfSe) tool. An LDA cut-off score of 2 or greater and unadjusted p-value threshold of 0.05 was used to report the significant findings.
(XLSX)

S6 Table LEFSe analysis and Differential abundance analysis in lactulose rhamnose ratio categories. **(A)** LEfSe (linear discriminant analysis Effect Size) analysis of gut microbiome in the study participants with high and low lactulose rhamnose ratio (LRR). **(B)** Differentially abundant microbial pathways (KEGG) predicted in study participants with high LRR using the linear discriminant analysis (LDA) Effect Size (LEfSe) tool. **(C)** Differentially abundant microbial enzymes (EC terms) predicted in study participants with high LRR using the linear discriminant analysis (LDA) Effect Size (LEfSe) tool. **(D)** Differentially abundant microbial MetaCyc pathways predicted in study participants with high LRR using the linear discriminant analysis (LDA) Effect Size (LEfSe) tool. An LDA cut-off score of 2 or greater and unadjusted p-value threshold of 0.05 was used to report the significant findings.
(XLSX)
