# Supplementary material for: Gut microbiota profiles of young South Indian children: Child sex-specific relations with growth
Source: PLoS One. 2021 May 14;16(5):e0251803. doi: 10.1371/journal.pone.0251803 (PMC8121364; doi:10.1371/journal.pone.0251803)

S1 Fig. Alpha-rarefaction curves. **(A)** Alpha-rarefaction curves showing observed OTU counts for all samples and samples grouped by **(B)** stunting status, **(C)** wasting status and **(D)** underweight status at different sample sequencing depths for maximum depth set as 60623 reads with 10 iterations per depth.

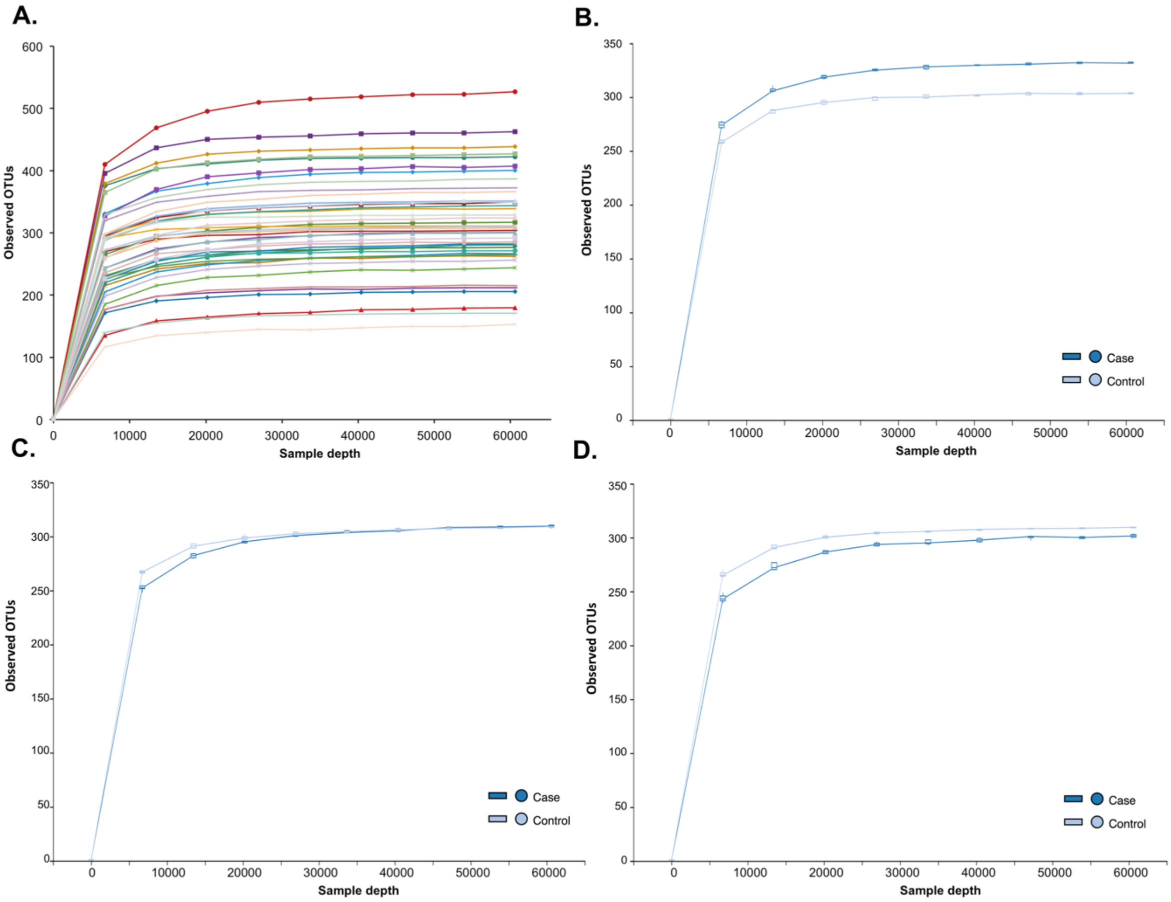

Supplement: S1 Fig — (A) Alpha-rarefaction curves showing observed OTU counts for all samples and samples grouped by (B) stunting status, (C) wasting status and (D) underweight status at different sample sequencing depths for maximum depth set as 60623 reads with 10 iterations per depth. (PDF) [file pone.0251803.s001.pdf]
