# Supplementary material for: Gut microbiota profiles of young South Indian children: Child sex-specific relations with growth
Source: PLoS One. 2021 May 14;16(5):e0251803. doi: 10.1371/journal.pone.0251803 (PMC8121364; doi:10.1371/journal.pone.0251803)

S2 Fig. Box plots of alpha diversity measures (Pielou's evenness, Faith's phylogenetic diversity index, Shannon diversity and observed OTUs) between cases and non-cases within stunting, wasting and undernutrition groupings. The outlier samples are highlighted in blue and yellow colors for cases and non-cases respectively.

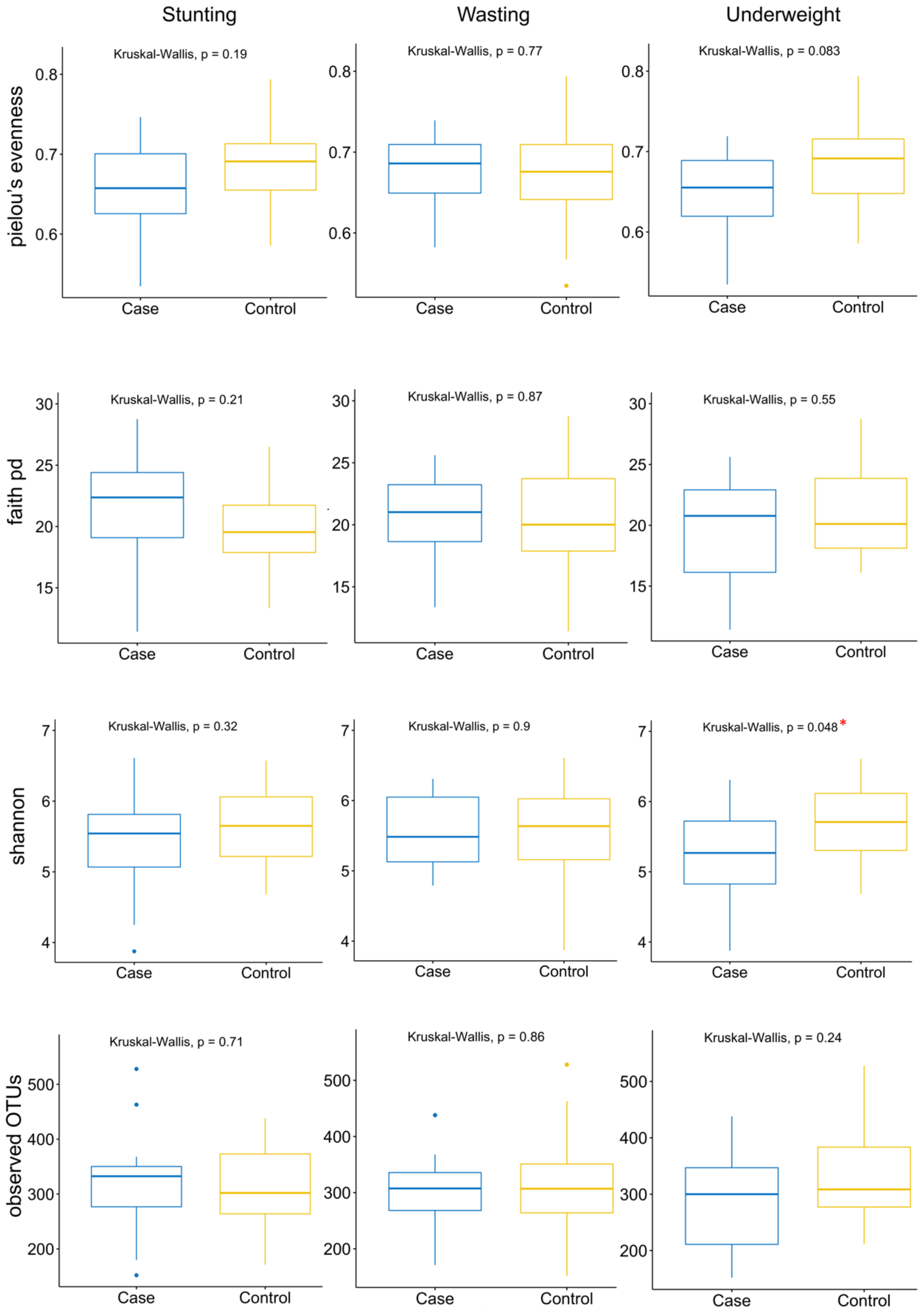

Supplement: S2 Fig — The outlier samples are highlighted in blue and yellow colors for cases and non-cases respectively. (PDF) [file pone.0251803.s002.pdf]
