# Supplementary material for: Gut microbiota profiles of young South Indian children: Child sex-specific relations with growth
Source: PLoS One. 2021 May 14;16(5):e0251803. doi: 10.1371/journal.pone.0251803 (PMC8121364; doi:10.1371/journal.pone.0251803)

S3 Fig. Emperor PCoA plots showing the clustering of samples based on beta diversity measures (Bray-Curtis, Jaccard's, unweighted and weighted UniFrac distances). Each dot represents a sample. P-values indicate group significances comparing cases and non-cases within stunting, wasting and undernutrition groupings using PERMANOVA. P<0.05 are represented in red font.

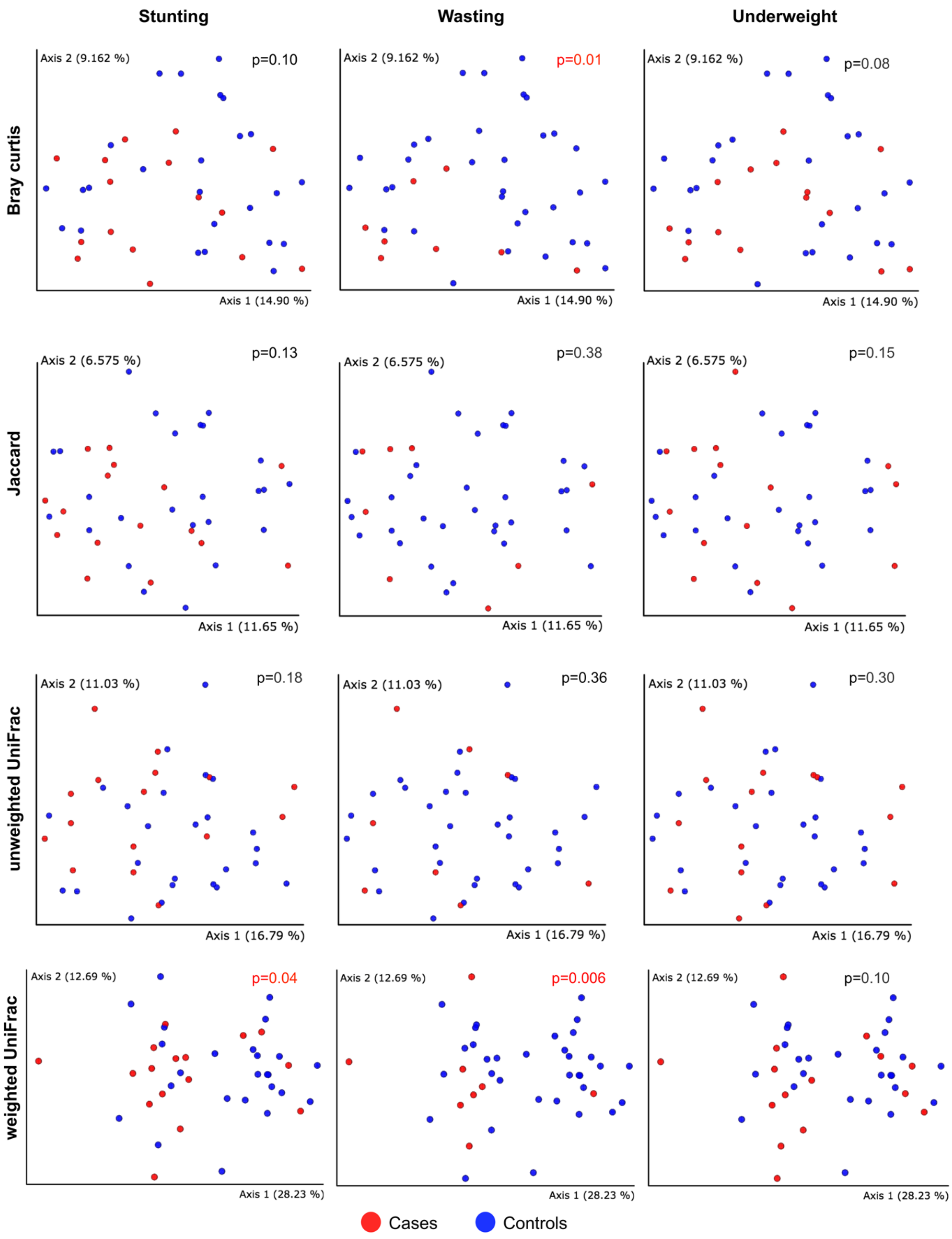

Supplement: S3 Fig — Each dot represents a sample. P-values indicate group significances comparing cases and non-cases within stunting, wasting and undernutrition groupings using PERMANOVA. P<0.05 are represented in red font. (PDF) [file pone.0251803.s003.pdf]
