# Supplementary material for: Gut microbiota profiles of young South Indian children: Child sex-specific relations with growth
Source: PLoS One. 2021 May 14;16(5):e0251803. doi: 10.1371/journal.pone.0251803 (PMC8121364; doi:10.1371/journal.pone.0251803)

S4 Fig. Scatter plots of association between fecal bacterial taxa and nutritional status of the study participants indicated by length-for-age (LAZ), weight-for-age (WAZ) and weight-for-length (WLZ) Z-scores and Hemoglobin and Age. Associations were determined using Spearman correlation (ρ). p = raw p-value, q = fdr-adjusted p-value.

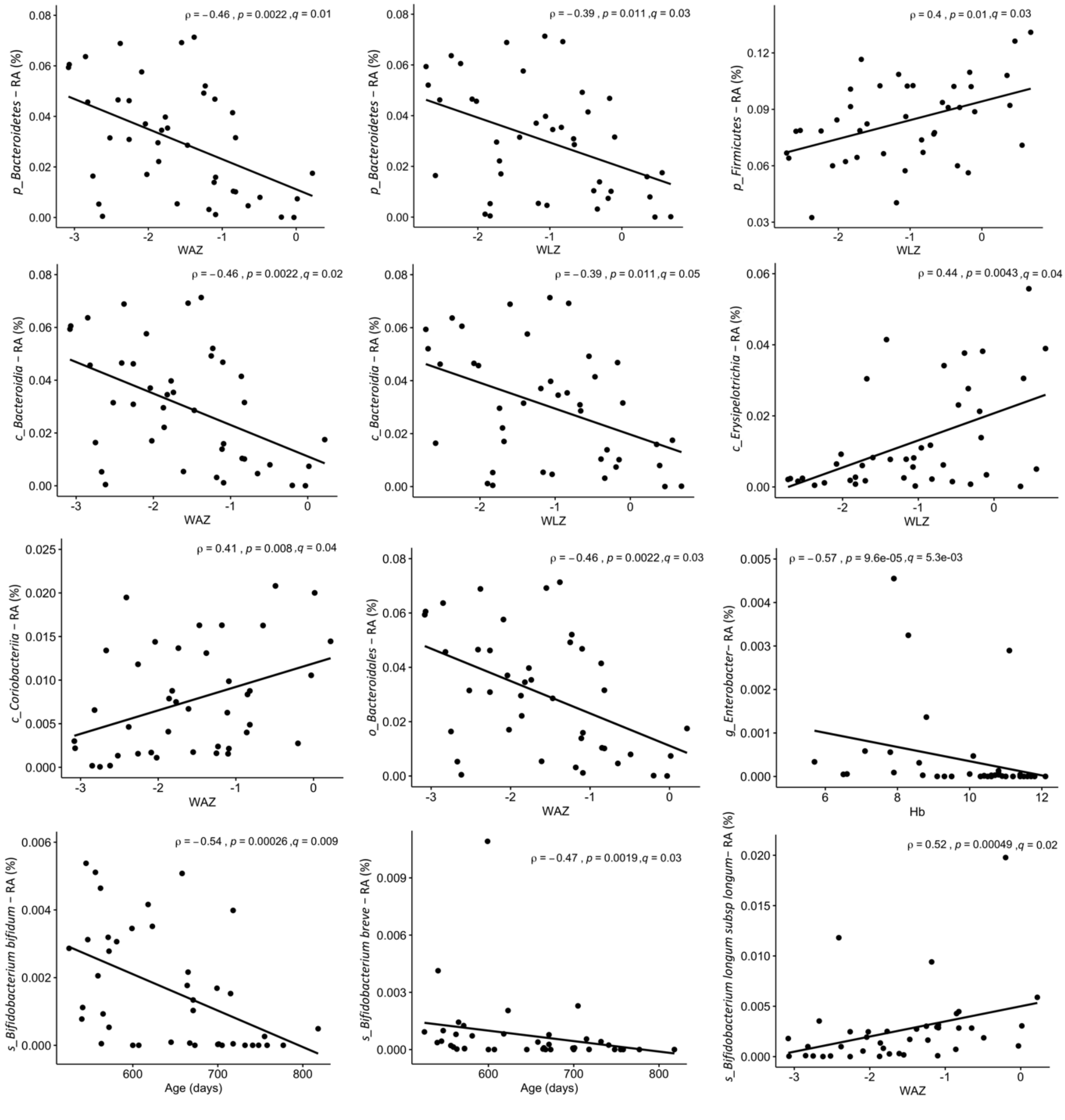

Supplement: S4 Fig — Associations were determined using Spearman correlation (ρ). p = raw p-value, q = fdr-adjusted p-value. (PDF) [file pone.0251803.s004.pdf]
