# Supplementary material for: Gut microbiota profiles of young South Indian children: Child sex-specific relations with growth
Source: PLoS One. 2021 May 14;16(5):e0251803. doi: 10.1371/journal.pone.0251803 (PMC8121364; doi:10.1371/journal.pone.0251803)

S5 Fig. A summary of differential abundant microbial enzymes (EC terms) predicted in stunted, wasted and undernourished children using the linear discriminant analysis (LDA) Effect Size (LEfSe) tool. An LDA cut-off score of 2 or greater and unadjusted p-value threshold of 0.05 was used to report the significant findings.

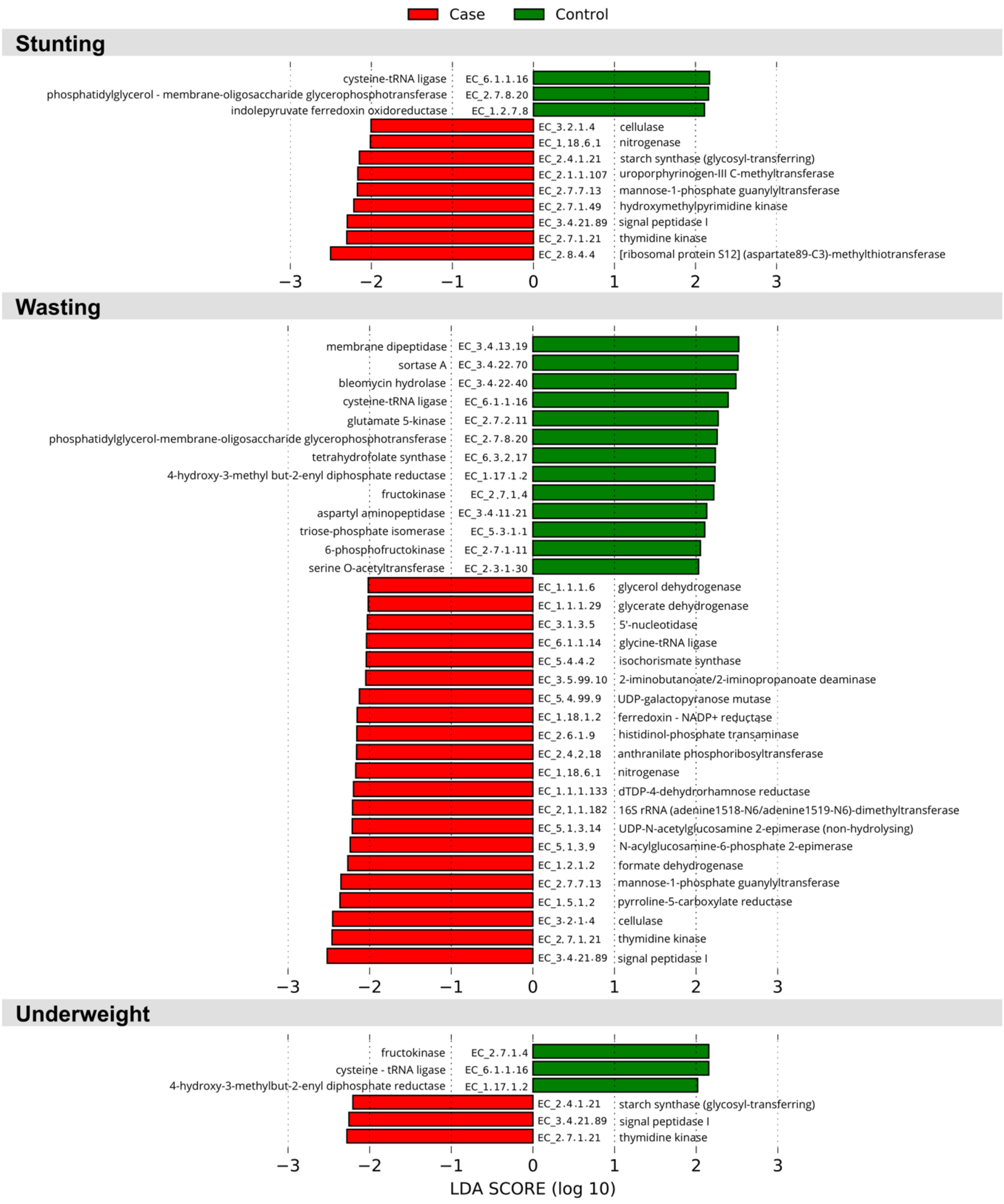

Supplement: S5 Fig — An LDA cut-off score of 2 or greater and unadjusted p-value threshold of 0.05 was used to report the significant findings. (PDF) [file pone.0251803.s005.pdf]
