# Supplementary material for: Gut microbiota profiles of young South Indian children: Child sex-specific relations with growth
Source: PLoS One. 2021 May 14;16(5):e0251803. doi: 10.1371/journal.pone.0251803 (PMC8121364; doi:10.1371/journal.pone.0251803)
Supplement: S1 Table — (PDF) [file pone.0251803.s008.pdf]

S1 Table. Data preprocessing statistics showing input raw read counts, final read counts after pre-processing and observed operational taxonomic units (OTUs) for each subject.

Sample-ID	Raw Read Count	Non-Chimeric Post QC Reads	Post Feature-Filtering Reads	Final OTU Count
EED-KT-041	1284174	163221	144787	210
EED-KT-042	1468049	126198	110471	466
EED-KT-043	1340705	122822	115257	353
EED-KT-044	1261600	176254	122330	267
EED-KT-045	1494658	140769	134720	280
EED-KT-046	1272706	103515	89462	423
EED-MB-011	1296492	206992	197757	288
EED-MB-012	955131	121253	108127	214
EED-MB-013	1083024	135597	120628	307
EED-MB-015	1035226	144723	130314	442
EED-MB-017	1100309	163250	158082	321
EED-MB-018	1166279	213111	204939	287
EED-MB-019	1038411	188207	179885	273
EED-MB-020	1245620	208433	198972	307
EED-MB-021	1284511	189887	181572	534
EED-MB-022	1108218	114882	111514	341
EED-MB-024	1205137	139344	134609	269
EED-MB-025	1019370	144975	137700	348
EED-MB-026	1171432	167105	165101	405
EED-MB-027	1367967	188951	186769	411
EED-MB-028	990817	177674	161097	184
EED-MB-029	800644	66258	60623	312
EED-MB-030	1164376	164941	155960	248
EED-MB-031	993065	126359	117646	273
EED-MB-032	997514	141533	134525	353
EED-MB-034	1064718	128336	119327	375
EED-MB-035	1074462	176203	166143	217
EED-MB-036	1068911	125416	119703	311
EED-MB-037	1125461	140001	122667	430
EED-MB-038	1155880	175517	170221	304
EED-MB-040	1081086	148579	137728	312
EED-MB-041	1217378	189863	181704	259
EED-MB-042	1053754	145428	135213	290
EED-MB-043	1139877	183896	170922	371
EED-MB-05	1227431	126891	117026	391
EED-MB-06	1247262	141970	127602	173
EED-MB-07	1240969	144012	129237	353
EED-MB-08	1332042	243487	235289	298

EED-RAGI-01	1045923	178921	173088	330
EED-RAGI-02	1035946	188679	178526	158
EED-RAGI-05	1107023	155778	125459	330
