# Supplementary material for: Gut microbiota profiles of young South Indian children: Child sex-specific relations with growth
Source: PLoS One. 2021 May 14;16(5):e0251803. doi: 10.1371/journal.pone.0251803 (PMC8121364; doi:10.1371/journal.pone.0251803)
Supplement: S2 Table — (PDF) [file pone.0251803.s009.pdf]

S2 Table. Characteristics of children categorised as cases and non-cases by nutritional status and lactulose rhamnose ratio*

	High LRR (LRR≥0.067)	Low LRR (LRR<0.067)	P-value
n	16	25	
Sex (F/M)	6/10	12/13	
Age (months)	22.2 ± 2.6	21.0 ± 2.6	0.166
Weight (kg)	9.5 ± 1.1	9.4 ± 1.2	0.840
Height (cm)	79.5 ± 3.6	79.7 ± 3.3	0.815
Maternal education (years)	8 (6.10)	10 (7,10)	0.095
Hb (g/dL)	9.8 (8.7,10.5)	10.8 (9.5,11.4)	(0.017)
%FFM ^	77 (73,78)	79 (75,83)	(0.198)
TEE (kcal/d)^	742 ± 178	791 ± 202	0.522
TEE (kcal/kg FFM/d) ^	105 ± 19	108 ± 26	0.783
LAZ	-1.9 ± 0.7	-1.4 ± 1.1	0.121
WAZ	-1.7 ± 0.6	-1.5 ± 1.0	0.577
WLZ	-1.0 ± 0.8	-1.1 ± 1.1	0.654
**Dual sugar assay**			
Rhamnose recovery (%)	1.4 (1.2,2.1)	1.5 (1.3,1.9)	(0.781)
Lactulose recovery (%)	0.19 (0.11,0.26)	0.06 (0.03,0.08)	(<0.001)
LRR	0.100 (0.089,0.145)	0.044 (0.029,0.052)	(<0.001)

*Values are mean ± SD and Median (Q1,Q3), P value for Independent t-test (Mann-Whitney test).
Abbreviation: HAZ, Height-for-age z-score; WAZ, Weight-for-age z-score; WHZ, Weight-for-Height z-score; Hb, Haemoglobin; %FFM, % fat free mass per kilogram body weight; TEE, Total Energy Expenditure; LRR, Lactulose Rhamnose ratio.
^n=10 and 20 for High and Low LRR respectively.
